# Supplementary material for: Conformational Plasticity of the Human Norovirus GII.3 Capsid Reveals Alternative P Domain Interaction Networks
Source: Int J Mol Sci. 2026 Jul 24;27(15):6586. doi: 10.3390/ijms27156586 (PMC13464932; doi:10.3390/ijms27156586)
Supplement: Supplementary file 1 [file ijms-27-06586-s001.zip › ijms-4426555-supplementary.pdf]

Supplementary information

Conformational plasticity of the human norovirus GII.3 capsid reveals alternative P domain interaction networks

Chihong Song[1,2,3], Motohiro Miki[4], Reiko Takai-Todaka[4], Kosuke Murakami[5], Kazuhiko Katayama[4*], Kazuyoshi Murata[6,7,8*]

[1] Department of Convergence Medicine, School of Medicine, Pusan National University, Yangsan 50612, Korea

[2] Core Research Facility, Pusan National University, Yangsan 50612, Republic of Korea

[3] Medical Research Institute, Pusan National University School of Medicine, Yangsan 50612, Korea

[4] Department of Infection Control Science and Immunology, Ōmura Satoshi Memorial Institute, Kitasato University, Tokyo, Japan

[5] Department of Diagnostic Testing and Technology Research, National Institute of Infectious Diseases, Japan Institute of Health Security, Shinjuku-ku. Tokyo 162-8640, Japan

[6] Exploratory Research Center on Life and Living Systems (ExCELLS), National Institutes of Natural Sciences, 38 Nishigonaka, Myodaiji, Okazaki, Aichi, 444-8585, Japan

[7] National Institute for Physiological Sciences, National Institutes of Natural Sciences, 38 Nishigonaka, Myodaiji, Okazaki, Aichi, 444-8585, Japan

[8] Department of Physiological Sciences, The Graduate University for Advanced Studies, Okazaki, Aichi, 444-8585, Japan

* Corresponding authors

E-mail: kazum@nips.ac.jp (Ka.M.)

E-mail: katayama@lisci.kitasato-u.ac.jp (K.K.)

Table S1. Data collection, image processing, and model statistics

Data collection	
Electron microscope	Titan Krios
Detector	Gatan K3
Accelerating voltage	300 kV
Magnification	53,000
Pixel spacing (Å)	1.35
Exposure time (s)	5.6
Electron dose rate	7.14 e^-/Å^2/s
Total electron dose	50 e^-/Å^2
Defocus range (μm)	0.5 - 2.5

Table S2. Image processing

Image processing				
Particle type	T = 3 (resting)		T = 3 (rising)	
Symmetry imposed	I1			
Number of micrographs	14,266			
Initial particle number	101,338			
Final particle number	8,498		18,996	
B-factor applied (Å^2)	-116		-604	
Global resolution (Å)	2.8		7.2	
EMDB number	EMD-81556		EMD-81567	
Focused refinement	A/B dimer	C/C dimer	A/B dimer	C/C dimer
Final particle number	150,531	62,943	-	432,078
Resolution (Å)	3.4	3.1	-	4.7
EMDB number	EMD-81571	EMD-81575	-	EMD-81576

Table S3. Model statistics

Model building	
Type of particles	T = 3 (resting)
Number of residues built	1541
RMSD (bonds)	0.006 Å
RMSD (angles)	0.904°
Ramachandran favored	96%
Ramachandran allowed	4%
Ramachandran outliers	0%
Rotamer outliers	0.15%
C-beta outliers	0
Clashscore, all atoms	5
PDB ID	27ZD

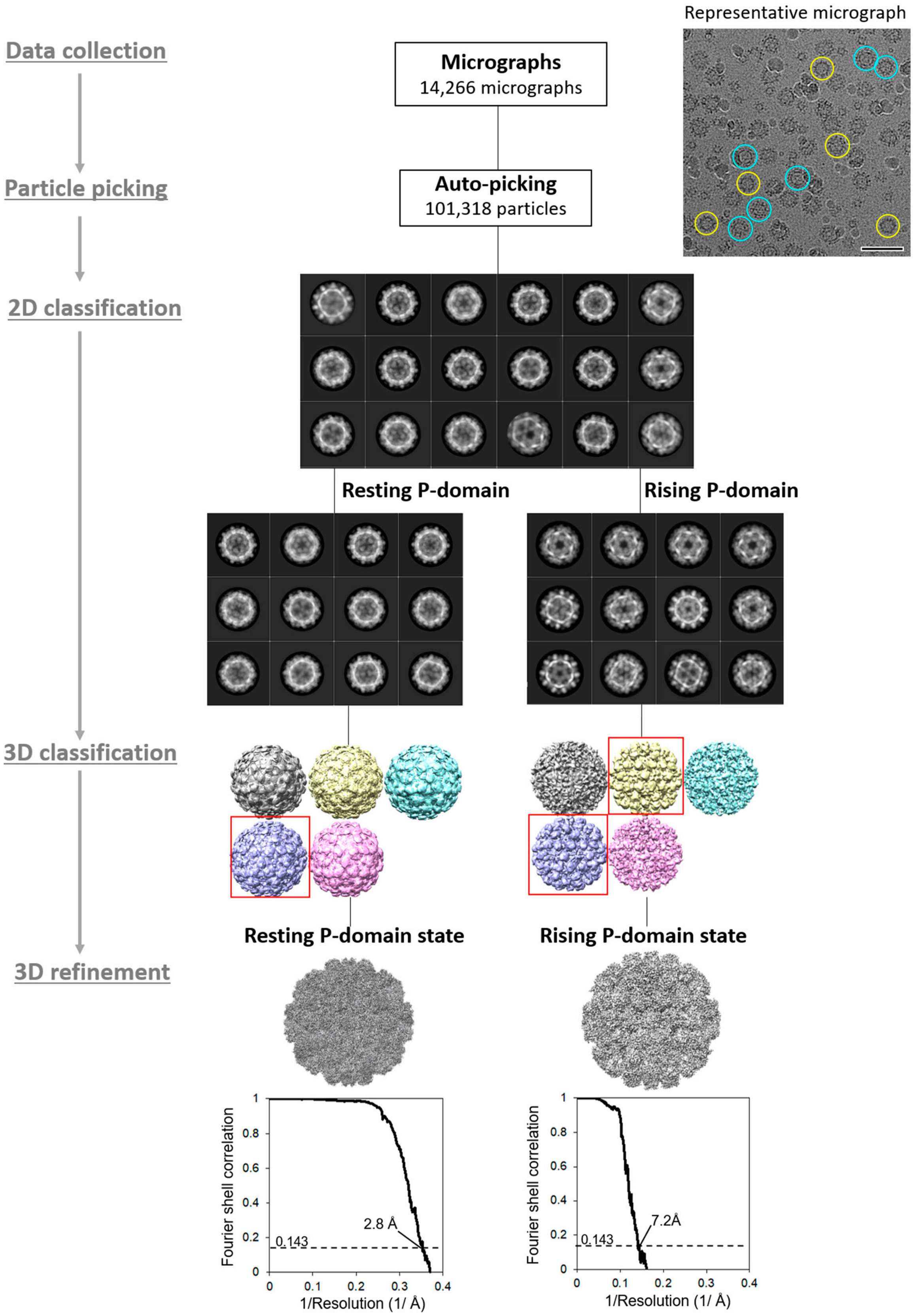

Data collection
Particle picking
2D classification
3D classification
3D refinement
Micrographs
14,266 micrographs
Auto-picking
101,318 particles
Representative micrograph
Resting P-domain
Rising P-domain
Resting P-domain state
Rising P-domain state
Fourier shell correlation
1/Resolution (1/ Å)
0.143
2.8 Å
7.2Å

Figure S1. Cryo-EM image processing workflow. The workflow for single-particle cryo-EM analysis of HuNoV GII.3 T = 3 VLPs is summarized. Particles were automatically picked from 14,266 micrographs and subjected to reference-free 2D classification. A total of 101,338 particles corresponding to T = 3 VLPs were refined and further processed through additional classification steps to remove low-quality particles and select homogeneous particle populations. The T = 3 particles were subsequently separated into resting- and rising state populations through an additional round of classification. Final 3D reconstructions were generated from 8,498 particles for the resting state and 18,996 particles for the rising state. Overall map resolutions were estimated using the gold-standard FSC criterion at an FSC threshold of 0.143, yielding resolutions of 2.8 Å for the resting state reconstruction and 7.2 Å for the rising state reconstruction. Corresponding FSC curves are shown at the bottom.

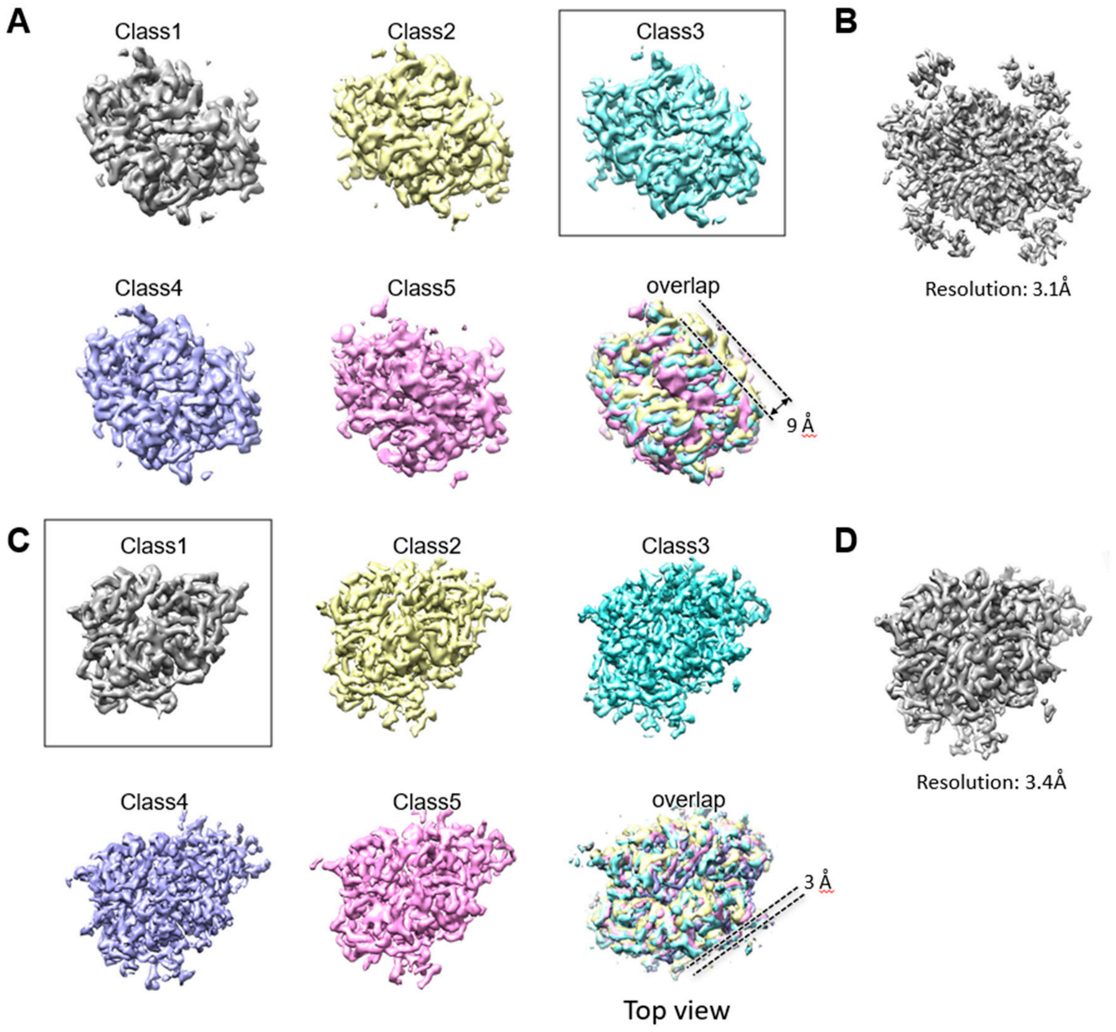

Figure S2. Focused refinement of the resting P domain dimers in the T = 3 particle. (A) Representative 3D classes of the resting state P domain in the C/C dimer obtained from symmetry-expanded particles by focused 3D classification without alignment. Because no alignment was performed during classification, each class retains the original spatial position of the corresponding C/C dimer within the T = 3 particle. The class selected for subsequent focused refinement is indicated by a box. The merged view illustrates positional variability of up to approximately 9 Å among the classified C/C dimer maps. (B) Focused-refinement reconstruction of the resting state P domain in the C/C dimer. The number of particles used for reconstruction

and the final resolution are indicated. (C) Representative 3D classes of the resting state P domain in the A/B dimer obtained from symmetry-expanded particles by focused 3D classification without image alignment. As no alignment was performed during classification, each class retains the original spatial position of the corresponding A/B dimer within the T = 3 particle. The class selected for subsequent focused refinement is indicated by a box. The merged view illustrates positional variability of up to approximately 3 Å among the classified A/B dimer maps. (D) Focused-refinement reconstruction of the resting state P domain in the A/B dimer. The number of particles used for reconstruction and the final resolution are indicated.

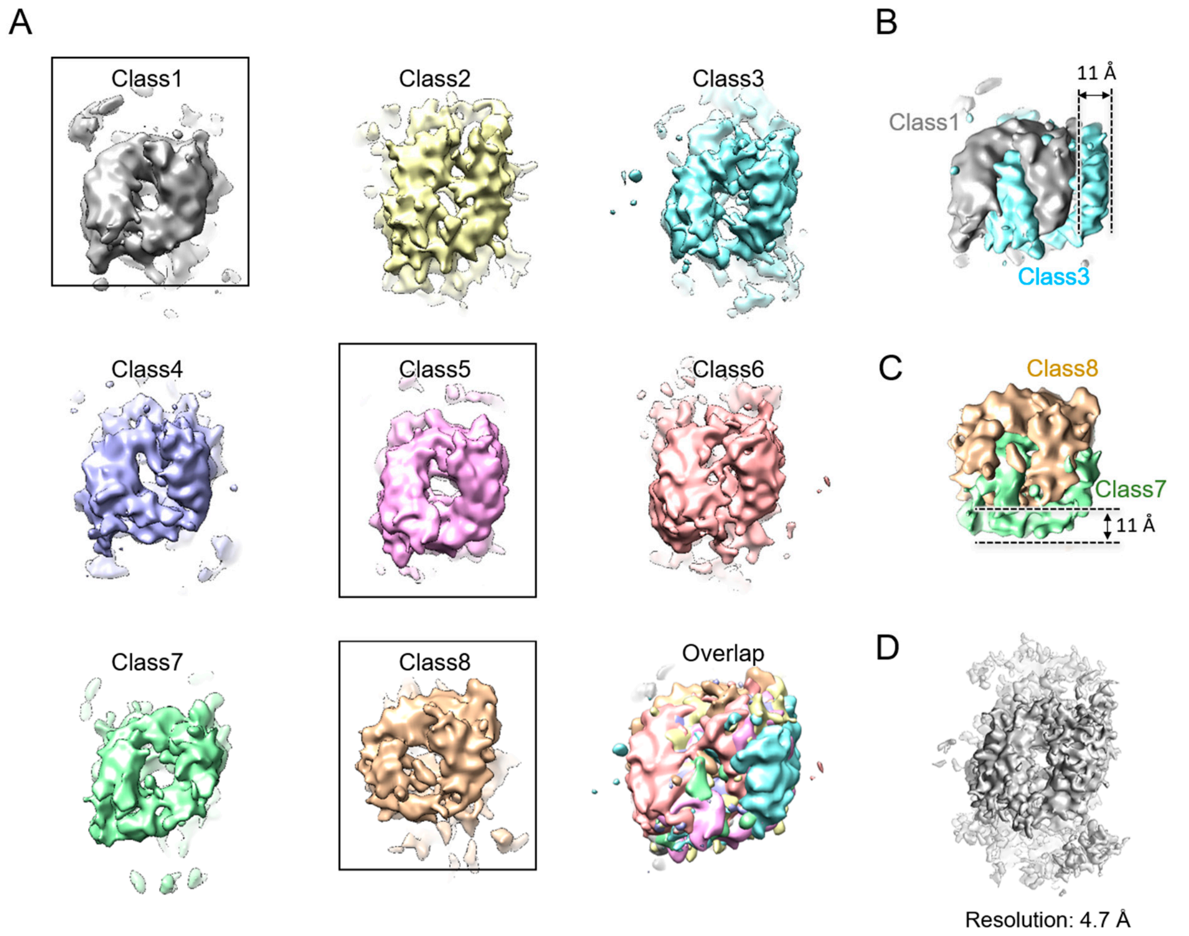

Figure S3. Focused refinement of the rising P domain C/C dimer in the T = 3 particle.

(A) Representative 3D classes of the rising P domain in the C/C dimer obtained from symmetry-expanded particles by focused 3D classification without alignment. Because no alignment was performed during classification, each class retains the spatial position of the corresponding C/C dimer within the T = 3 particle. Selected classes used for comparison are boxed. The merged view shows positional variability among the classified C/C dimer maps. (B, C) Superimposed views of selected 3D classes showing positional variability of up to approximately 11 Å in different directions. (D) Focused-refinement map of the rising P domain in the C/C dimer. The number of particles and resolution are indicated in the panel.
